# The goldfish *Carassius auratus*: an emerging animal model for comparative cardiac research

**DOI:** 10.1007/s00360-021-01402-9

**Published:** 2021-08-28

**Authors:** Mariacristina Filice, Maria Carmela Cerra, Sandra Imbrogno

**Affiliations:** Department of Biology, Ecology and Earth Sciences, University of Calabria, 87036 Arcavacata di Rende, CS Italy

**Keywords:** Goldfish heart, Hypoxia, Nitric oxide, Cardioactive peptides, BPA, Cardiac remodelling

## Abstract

The use of unconventional model organisms is significantly increasing in different fields of research, widely contributing to advance life sciences understanding. Among fishes, the cyprinid *Carassius auratus* (goldfish) is largely used for studies on comparative and evolutionary endocrinology, neurobiology, adaptive and conservation physiology, as well as for translational research aimed to explore mechanisms that may be useful in an applicative biomedical context. More recently, the research possibilities offered by the goldfish are further expanded to cardiac studies. A growing literature is available to illustrate the complex networks involved in the modulation of the goldfish cardiac performance, also in relation to the influence of environmental signals. However, an overview on the existing current knowledge is not yet available. By discussing the mechanisms that in *C. auratus* finely regulate the cardiac function under basal conditions and under environmental challenges, this review highlights the remarkable flexibility of the goldfish heart in relation not only to the basic morpho-functional design and complex neuro-humoral traits, but also to its extraordinary biochemical-metabolic plasticity and its adaptive potential. The purpose of this review is also to emphasize the power of the heart of *C. auratus* as an experimental tool useful to investigate mechanisms that could be difficult to explore using more conventional animal models and complex cardiac designs.

## Introduction

Since 1901, when Underwood (1901) first described the role of the goldfish *Carassius auratus* in destroying mosquito larvae, the use of this teleost in research considerably increased over the years. It now represents a largely used model organism in endocrinology (Blanco et al. 2018), cell biology (Choresca et al. 2009), immunology (Hanington et al. 2006), neurobiology (Portavella et al. 2004; Preuss et al. 2006), and ecotoxicology (Xia et al. 2013; Filice et al. 2020a). Of remarkable relevance for research is the extraordinary ability exhibited by the goldfish to face stressful conditions, as those experienced in both natural life and aquaculture. It can tolerate wide pH variations (Szczerbowski 2001), high levels of turbidity (Wallen 1951), and temperature fluctuations (Ford and Beitinger 2005). The goldfish shares with other members of the cyprinid genus *Carassius* (e.g., the crucian carp *Carassius carassius*) the striking capacity to survive and remain active for long periods under hypoxia, even tolerating anoxia (Bickler and Buck 2007). This is correlated to a strong metabolic depression (to approximately 30% of normal) that allows to maintain muscle and liver glycogen stores, and to the capacity to escape acidosis by converting lactate to ethanol and CO_2_, excreted through the gills (Shoubridge and Hochachka 1980).

A fascinating trait of cyprinids is their ability to preserve [*C. carassius*: (Stecyk et al. 2004)], or even improve [*C. auratus*: (Filice et al. 2020b; Imbrogno et al. 2014, 2019a; Leo et al. 2019)] the cardiac performance when challenged by protracted hypoxia/anoxia. This was proposed to importantly contribute to adapt the physiological interactions between organs and tissues, under the challenge of a reduced O_2_ (Imbrogno et al. 2018; Gattuso et al. 2018). Accordingly, the cyprinid heart is considered a useful tool to investigate the mechanisms that allow survival by maintaining the cardiac function under low O_2_.

In the case of the goldfish other advantages can be considered. One is its size that makes it easy for maintenance and handling, allows tissue isolation and collection, as well as in vivo, ex vivo and in vitro cardiac functional studies (Blanco et al. 2018). The electrical properties of the goldfish heart resemble that of larger mammals, and this makes it attractive also for mammalian-oriented translational studies. Examples are the in vivo values of heart rate of 111 beats/min in fish acclimated at 20 °C (Ferreira et al. 2014), and the duration and kinetics of the epicardial action potential (AP) and Ca^2+^ transients, which are similar to those reported for humans and dog endocardial APs (Bazmi and Escobar 2020). The research possibilities offered by this cyprinid are further expanded by experimental evidence showing that several hormones and peptides, acting in mammals as cardiac modulators (Rocca et al. 2018a, 2019b; Angelone et al. 2015; Filice et al. 2015), also influence the heart performance of *C. auratus* (Imbrogno et al. 2014, 2017; Mazza et al. 2015; Leo et al. 2019) under basal conditions and environmental challenges. The picture emerging from the available information has recently stimulated attention toward the goldfish, with the purpose of uncovering the mechanisms that provide high plasticity to the heart, particularly under internal and external challenges.

This review is aimed to provide an overview of the regulatory mechanisms that, in the goldfish, control the cardiac performance under basal conditions and in the presence of challenges, with particular focus on the mechanisms activated by intrinsic (hemodynamic loads), extrinsic (nervous and humoral control), biotic (temperature, hypoxia), and abiotic (water pollutants) factors. When possible, literature data will be discussed by taking into consideration the remarkable functional flexibility of the goldfish heart in response to low O_2_. To facilitate the reader unfamiliar with the goldfish heart, a brief outline of the cardiac structural and functional traits will be first provided.

## Cardiac morphological design

**Fig. 1:**
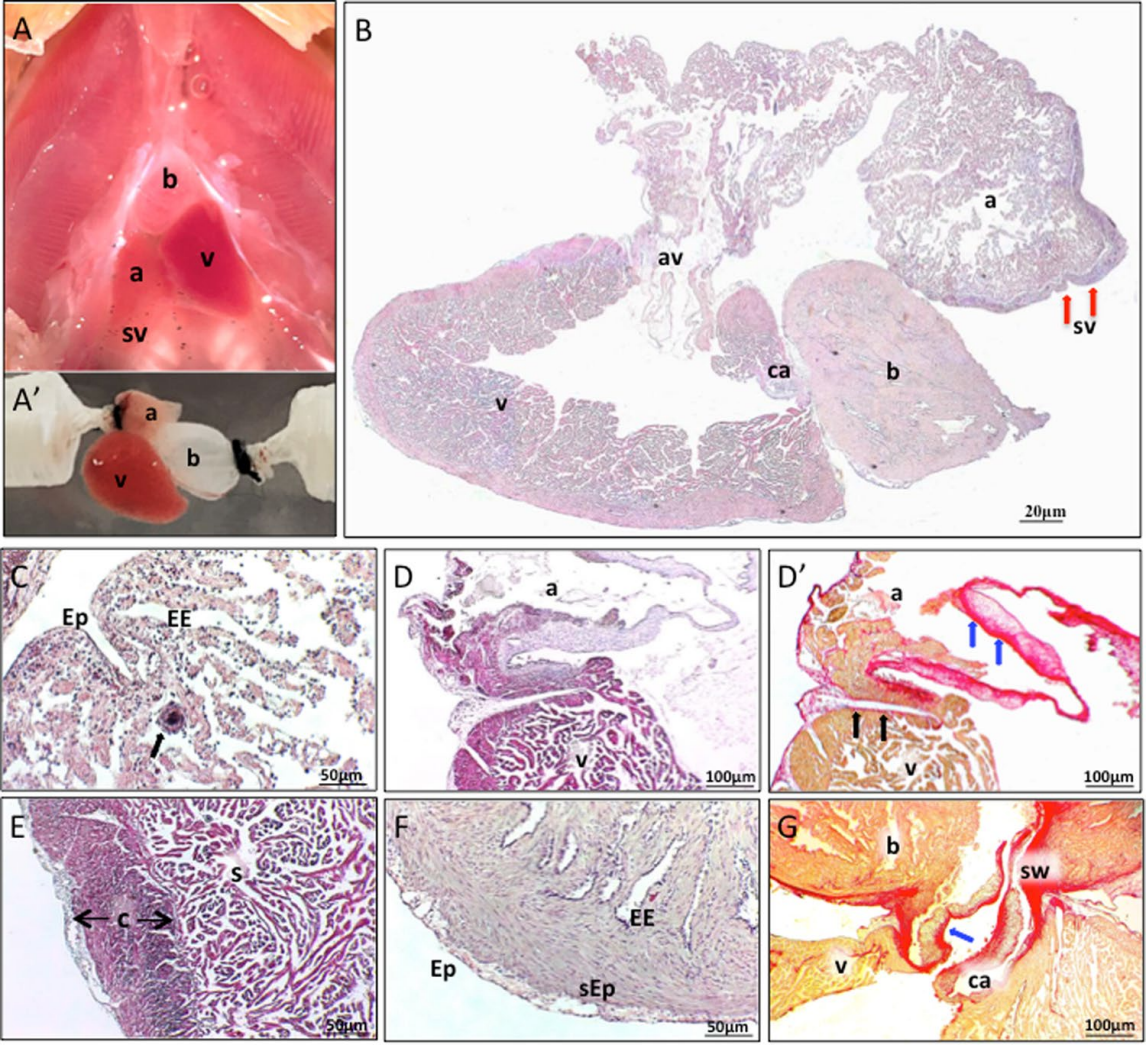
Photographs showing the anatomical organization of in situ (**A**) and isolated goldfish heart (**A’**); **B** sagittal section of the whole heart stained with hematoxilin–eosin. *a* atrium, *b* bulbus, *v* ventricle, *sv* sinus venosus, *av* atrio-ventricular region, *ca* conus arteriosus. **C–G** Structural features of heart regions. **C** Hematoxylin–eosin stained section of atrium; a detail of a terminal nerve ending is evident (black arrow); *Ep* epicardium, *EE* endocardium. (**D–D’**) Longitudinal section of the atrio-ventricular region stained with hematoxylin–eosin (**D**) and Sirius Red (**D’**): the connective ring surrounding the vascularized muscular tissue (black arrows) and the thick fibrosa of the atrio-ventricular valves (blue arrows) are indicated. **E** Mallory's trichrome stained ventricular section showing *compacta* (c) and *spongiosa* (s). **F** Hematoxilin–eosin longitudinal section of the bulbus; *Ep* epicardium, *sEp* sub-epicardium, *EE* endocardial endothelium. **G** Sirius red staining of the conus arteriosus showing the high collagen amount (blue arrow) in the thick fibrosa (ventricular layer of the conus valve leaflets). **B**, **D’**, **G**: modified from (Garofalo et al. 2012); **E**: modified from (Imbrogno et al. 2019b); **C**, **D**, **F**: D. Amelio unpublished data

**Fig. 2:**
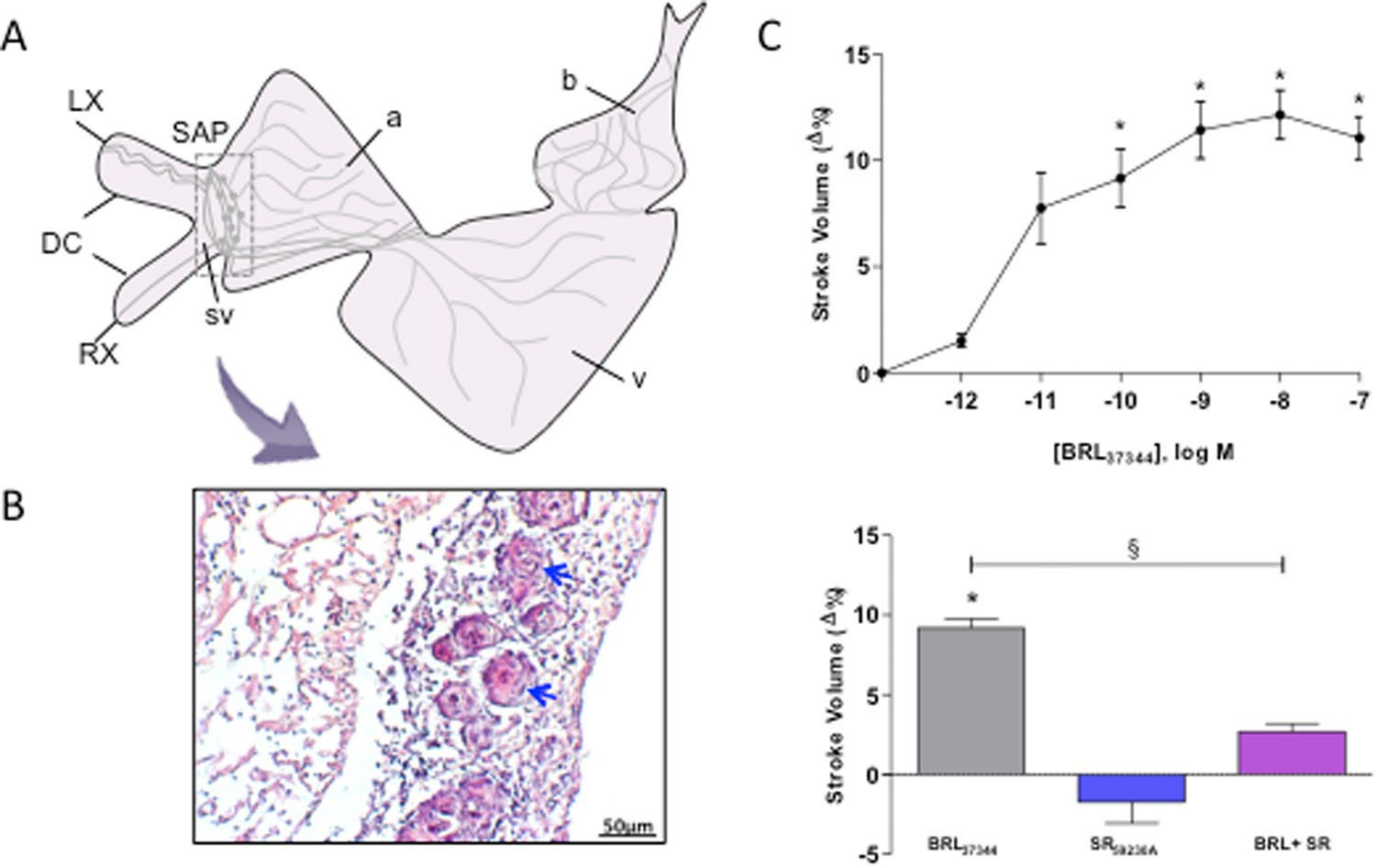
**A** Schematic representation of the general innervation pattern of the goldfish heart [modified from (Newton et al. 2014)]. Ducts of Cuvier (DC), and right (RX) and left (LX) vagosympathetic trunks are showed. *a* atrium, *b* bulbus, *SAP* sinoatrial plexus, *sv* sinus venosus, *v* ventricle. **B** Hematoxylin–eosin stained section of the sinus venosus wall containing rings (blue arrows) of nervous tissue corresponding to the primitive pacemaker region [modified from (Garofalo et al. 2012)]. **C** Effects of adrenergic stimulation in the isolated and perfused working goldfish heart. Upper panel: cumulative dose–response curve of the β3-AR-specific agonist, BRL_37344_, on stroke volume (*p < 0.05: BRL vs. control); lower panel: effects of BRL_37344_ (10^–9^ M) on stroke volume before and after treatment with the specific β3-AR antagonist, SR_59230A_ (10^–8^ M) (*p < 0.05: BRL vs. control; ^‡^*p* < 0.05: BRL vs. BRL + SR) [modified from (Leo et al. 2019)]

The heart of *C. auratus* consists of four chambers, i.e., the *sinus venosus*, the atrium, the ventricle, and the bulbus arteriosus; it also includes two distinct structures corresponding to the atrioventricular (AV) region and the conus arteriosus (Fig. 1A, B) (Garofalo et al. 2012). The *sinus venosus* represents the most caudal portion of the heart functionally related to a *vis-a-tergo* atrial filling. In the goldfish, it consists of a thin wall of connective tissue and shows, in the proximity of the sino-atrial (SA) region, rings of nervous tissue resembling ganglion cells which are proposed as a primitive pacemaker (Yamauchi 1980) (Fig. 2B). The atrium is a large chamber delimited by a rim of myocardium enveloping a complex network of thin trabeculae surrounded by elastin and collagen fibres (Garofalo et al. 2012). In teleost, the AV region, a distinct species-specific cardiac segment, connects the atrium with the ventricle (Icardo and Colvee 2011). In the goldfish, it consists of a ring of vascularized compact myocardium surrounded by connective tissue formed by loose collagen fibres (Fig. 1D, D’) (Garofalo et al. 2012). This ventricular structural organization, typical of the approximative one-third of teleost species, including the goldfish, consists of both a vascularized *compacta* and a trabeculated *spongiosa *(Fig. 1E) (Tota et al. 1983; Farrell and Jones 1992). The thin *compacta*, localized in the outer region of the ventricular wall, is formed by bundles of muscle tissue variously oriented and highly vascularized by coronary vessels. The underlying *spongiosa* is avascular and contains numerous trabeculae covered by a thin layer of endocardial endothelium (EE) cells and connected by endocardial bridges. With respect to the most active teleosts (e.g. tuna), in which a thicker *compacta *provides the potential to act as pressure pumps (i.e., they move small volumes at a relatively high rate and high pressure) (Tota and Gattuso 1996; Icardo et al. 2005), the goldfish heart is able to move large stroke volumes (*V*s) at a low heart rate, being not able to produce high pressures; thus, it functions as a volume pump heart (Garofalo et al. 2012). A layer of collagen fibres, located at the boundary between *compacta* and *spongiosa*, functions as an anchorage structure which mechanically link the two differently oriented muscular layers (Icardo et al. 2005). This maintains ventricular structure and dynamics and guarantees the functional synchronism between the *compacta* and the *spongiosa*, necessary to prevent ventricular dyssynergy (Imbrogno 2013).

The outflow tract is characterized by two structures: the proximal *conus arteriosus* (Fig. 1G), which provides support to the valve complex (Schib et al. 2002), and a distal *bulbus arteriosus* (Fig. 1F). The bulbar wall is organized into layers; moving from the lumen, they consist of the endocardium, the endocardial ridges, the middle layer, the subepicardium and the epicardium (Fig. 1F). Endocardial cells cover the columnar ridges, while the middle layer encloses loose smooth muscle cells interposed between abundant elastin and few collagen fibres. Similar to other teleosts [e.g., carp: (Licht and Harris 1973); trout: (Serafini-Fracassini et al. 1978)], the bulbus chamber contains a large amount of elastin which makes it extremely flexible, thus allowing the elastic recovery [windkessel effect; (Farrell and Jones 1992)] which ensures a constant blood flow towards the gills. At the same time, the high levels of collagen fibres prevent an excessive expansion of the bulbus during ventricular systole.

As for other teleosts, the goldfish heart receives a dual vagal autonomic innervation (Newton et al. 2014): the cardiac vagal rami carry both cholinergic and adrenergic innervation, indicating that also in this species the vagus is a vagosympathetic trunk (Nilsson 2011; Laurent et al. 1983). Nerves enter the heart by coursing along the wall of the sinus venosus toward the SA junction where they interact with intracardiac neurons (ICN) to form a dense SA plexus (SAP) (Fig. 2A). The majority of axons entering the SAP are cholinergic, although few tyrosine hydroxylase (TH) containing axons, mainly deriving from spinal autonomic postganglionic inputs, are also identified (Newton et al. 2014). In teleosts, the SA region is the site of the cardiac pacemaker. This is demonstrated by extensive physiological evidence showing, at the base of the sinoatrial valves (SAV), pacemaker-type electrical activity, and the presence of molecular pacemaker markers, such as the sodium/potassium hyperpolarization-activated cyclic nucleotide-gated channel (HCN4), and the transcription factor, Islet-1 (Haverinen and Vornanen 2007; Sedmera et al. 2003; Tessadori et al. 2012; Vornanen et al. 2010). In the goldfish, a population of cells expressing HCN4 immunoreactivity, innervated by numerous ICN, is present in the basal SAV region (Newton et al. 2014), implying a local neural circuit for the autonomic modulation of pacemaker discharge rate and the related cardiac output (Newton et al. 2014). From the SAP, nerve fibres enter the atrial wall bilaterally: the largest reaching the AV canal and the ventricle, and others diverging to innervate myocytes in atrial trabeculae (Newton et al. 2014). Both choline acetyltransferase (ChAT)- and TH-positive axons innervate the atrium, although with a higher cholinergic than adrenergic content. In teleosts, the adrenergic innervation of the atrium is well characterized, while the presence of a cholinergic innervation remains controversial, being apparently lacking in some species or predominant in others [reviewed by (Laurent et al. 1983; Gibbins 1994)]. It has been postulated that in the goldfish, the dual innervation of the atrium allows a fine modulation of its contractility (Newton et al. 2014). Innervation in the AV canal forms a plexus at the atrioventricular junction, which circumscribes the AV valves and makes contact with a small population of ICN. Although its function remains unknown, the identification of a secondary pacemaker at the AV junction in the carp (Saito and Tenma 1976), as well as the presence of myocytes in the AV valve leaflets of the goldfish (Newton et al. 2014) suggest a role in controlling either local pacemaker rate or valve function. In the goldfish, as in the majority of teleosts, the ventricle, which is a type II ventricle, is innervated by both cholinergic and adrenergic axons. Cholinergic axons target both coronary blood vessels and cardiomyocytes of the compact myocardium, while adrenergic axons primarily innervate coronary vessels (Newton et al. 2014). This distribution suggests that in these species, the ventricle is under a dual autonomic control, with a cholinergic innervation that primarily modulates contractility and an adrenergic control that mainly affects coronary blood flow (Newton et al. 2014).

## Regulation of the cardiac performance

In fishes, as in other vertebrates, the heart shows a remarkable ability to regulate its pumping performance to match the variable hemodynamic requirements. Under normal conditions, cardiac output (CO) is mainly determined by the interplay between myocardial contractility, vascular conductance (resistance) and capacitance (mostly determined by the venous circulation) (Joyce and Wang 2020). Frequency (HR) and *V*s modulation are both effective strategies for regulating CO; however, their relative contribution varies substantially among fish species also in relation to different types of challenge. For example, a postprandial increase in HR, rather than in *V*s, has been reported in rainbow trout (Grans et al. 2009). Moreover, although during exercise salmonids regulate CO by significantly increasing *V*s [for extensive review see (Farrell et al. 2009)], data from polar, temperate and tropical species propose a predominant role of HR in regulating CO during swimming (Axelsson et al. 1992; Clark and Seymour 2006; Iversen et al. 2010), and in response to increased oxygen demand (Farrell 2016; Eliason and Anttila 2017), questioning the predominant dogma that, during exercise, fish regulate *V*s more than HR (Joyce and Wang 2020).

As in other teleosts, in the goldfish the heart performance is regulated by a number of interplaying mechanisms, only partially explored in this species. As hereafter reported, hemodynamic loads (filling pressure or preload and output pressure or afterload) are key determinants of CO. At the same time, the goldfish heart is a target of a rich extrinsic modulation achieved via neuro-humoral circuits that includes adrenergic and cholinergic innervation, endogenous cardioactive substances (i.e., the chromogranin-A-derived peptides, nesfatin-1, SELENOT), and locally released autocoids (e.g., nitric oxide), whose modulatory role is here following discussed.

### Intrinsic regulation: the Frank–Starling mechanism

The Frank–Starling mechanism is a fundamental property of the heart of all vertebrates. It allows the myocardium to respond to increased filling pressure with a more vigorous contraction of its lengthened fibres. In mammals, the cardiac response to preload is characterized by a biphasic module: an immediate increase in the force (i.e., the heterometric regulation or the Frank–Starling response), followed, after 10–15 min, by a slower and persistent increment of force (the Anrep effect). The first stretch-related increase in developed force is correlated with a length-dependent increase in cross-bridge formation and myofilament calcium responsiveness (Katz 2002), while the subsequent slow increase is attributed to an increase in the intracellular Ca^2+^ transient (Calaghan et al. 1999; Casadei and Sears 2003). In fish, it is not yet clear whether the cardiac response to stretch assumes the same biphasic module, however, it is commonly recognized that the end-diastolic volume and the consequent stretch-related increase in developed force is a major regulator of the cardiac performance (Olson 1998). Teleosts show an elevated sensitivity to the Frank-Starling response [as reviewed in (Imbrogno et al. 2018; Shiels and White 2008; Olson 1998)]; this has been attributed to a great myocardial extensibility of the highly trabeculated heart, coupled to a maintained increase in myofilament Ca^2+^ sensitivity over a large range of sarcomere lengths (Shiels and White 2008). Moreover, growing evidence suggests that, as in mammals [see for example, (Massion et al. 2005; Zhang et al. 2008)], in fish the Frank–Starling response is modulated by autacoids and humoral factors. For example, in the eel *Anguilla anguilla,* the beat-to-beat regulation of the in vitro working heart involves nitric oxide (NO) that modulates ventricular relaxation by involving phospholamban (PLN) S-nitrosylation-dependent Ca^2+^ reuptake by SERCA2a pumps (Garofalo et al. 2009). Similar to other vertebrates, the fish heart also shows the intrinsic ability to maintain resting *V*s over a broad range of physiological output pressures (i.e., the homeometric regulation) (Farrell and Jones 1992). This range is species-specific and correlates with in vivo ventral aortic pressure; i.e., hearts from more active fishes better sustain higher diastolic afterload and have higher ventral aortic pressures [see (Farrell 1991)]. This occurs up to a certain output pressure, beyond which homeometric regulation breaks down, the heart is unable to completely empty, leading to increased end-diastolic volume and reduced *V*s (Farrell 1991).

**Fig. 3:**
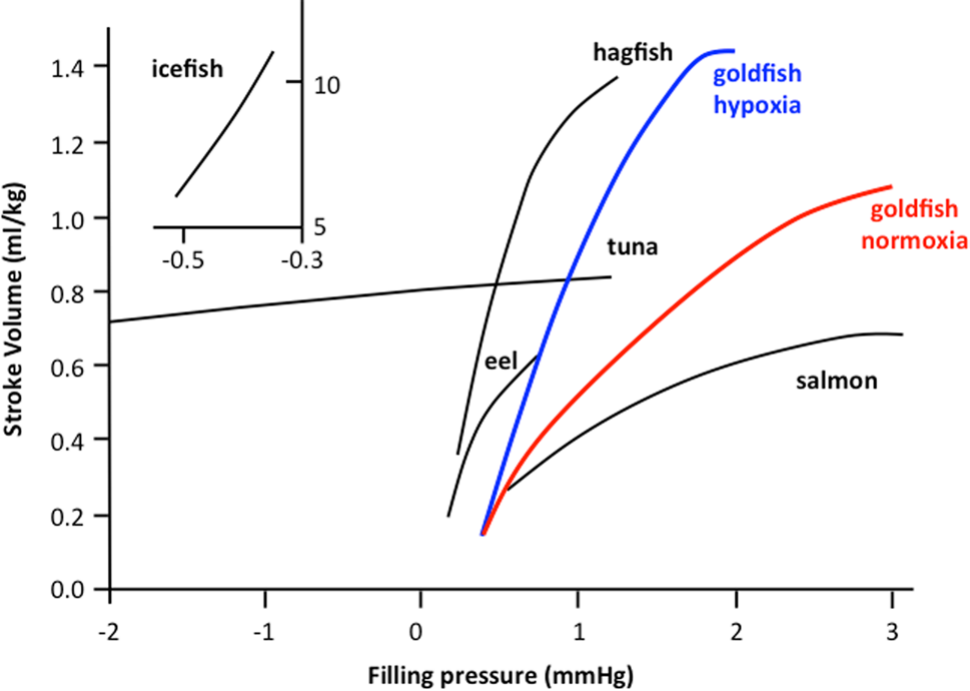
Starling curves obtained in in vitro hearts of several fish. Compared to other teleost species sharing a type II ventricle (e.g., salmon), the goldfish heart shows a marked sensitivity to preload increases, reaching stroke volume values of about 1.08 mL/kg under normoxia (red line) and 1.45 mL/kg under acute hypoxia (blue line). Modified from (Olson 1998)

The heart of *C. auratus* is a typical volume pump. Under basal conditions, CO values [11.85 mL/min/kg at 18 °C (Garofalo et al. 2012)] are similar to those of other teleosts [e.g., crucian carp: 8.4 mL/min/kg at 8 °C (Farrell and Stecyk 2007); European eel: 11.8 mL/min/kg (Peyraud-Waitzenegger and Soulier 1989), 10 mL/min/kg at 18 °C (Imbrogno et al. 2001); rainbow trout: 15 mL/min/kg at 12 °C (Agnisola et al. 2003)]. It shows a marked sensitivity to filling pressure changes, as revealed by the elevated *V*s values (~ eightfold increase) reached in response to increasing preloads (Garofalo et al. 2012). This hemodynamic ability to move high blood volumes at little input pressures is shared by fish characterized by highly trabeculated ventricle, as in the case of the icefish and of salmonids (Fig. 3). However, similarly to its relative crucian carp (Stecyk et al. 2004), and in contrast with other temperate teleosts, which show the ability to sustain values of afterloads up to 5 kPa (Farrell and Jones 1992; Tota and Gattuso 1996), in the goldfish, afterload pressures higher than 1.5 kPa significantly reduced cardiac performance (Garofalo et al. 2012).

The ability to displace high blood volumes at low pressures is supported by structural requirements such as a high atrial/ventricular relative mass (27.6%), associated with lacunary spaces in the *spongiosa* (i.e., the lacunae which separate the trabeculae of the *spongiosa*) of both the atrium and the ventricle (Garofalo et al. 2012). As discussed later in the review, this hemodynamic behaviour is important for the cardiac response to hypoxia.

Of relevance, in the goldfish, the cardiac response to filling pressure appears to be related to animal body weight (Filice et al. 2020a). It has been reported that animals with small size (body weights ranging between 25 and 40 g) at their maximum *V*s have a higher filling pressure [*V*s: 1.13 ± 0.13 mL/kg^−1^ body mass; input pressure: 0.95 kPa (Filice et al. 2020a)] with respect to those with larger size [body weights ranging between 45 and 95 g; *V*s: 1.08 ± 0.09 mL kg^−1^ body mass; input pressure: 0.4 kPa (Garofalo et al. 2012; Imbrogno et al. 2014)]. These data, which are in line with observations in the European eel showing that changes in cardiac performance correlate with growth-dependent differences in animal size (Cerra et al. 2004), are probably related to age-related characteristics of the morpho-structural design of the goldfish ventricular pump (Cerra et al. 2004). An aspect that deserves future investigations.

Very recently, an exhaustive picture of cardiac electrophysiology and Ca^2+^ dynamics in perfused intact goldfish heart has been provided by Bazmi and Escobar (2020). They showed that, at room temperature, the goldfish ventricular APs duration is longer than those measured in zebrafish (goldfish APD_90_: 481.5 ± 9.5 ms vs. zebrafish APD_90_: 136.5 ± 9.0 ms) and is more similar to that reported in the endocardial ventricular layer of dog heart (Piktel et al. 2011). Likewise, goldfish Ca^2+^ transients are slower than those in the zebrafish (Bazmi and Escobar 2020), while kinetic characteristics are similar to those reported for canine hearts (Belevych et al. 2011; Laurita et al. 2003). Of note, the fraction of Ca^2+^ released from the SR, which contributes to the amplitude of the systolic Ca^2+^ transient, is similar between goldfish and larger mammals (Belevych et al. 2011).

### Extrinsic regulation: the autonomic nervous control

In the majority of teleosts, the heart function is extrinsically modulated by a parasympathetic (cholinergic) and a sympathetic (adrenergic) innervation which reaches the heart via the vagus nerve (Laurent et al. 1983; Nilsson 1983). As extensively described by Newton et al., and anticipated above in the text, also the goldfish heart receives a dual vagal autonomic innervation, with cholinergic and adrenergic axons targeting all chambers of the heart. Positive ChAT and TH immunostaining are present in intracardiac neurons mainly located in the large plexus surrounding the SAV (Newton et al. 2014), indicating a cholinergic and adrenergic regulation (Newton et al. 2014). Studies in various fish species from polar, temperate and tropical areas [reviewed in (Sandblom and Axelsson 2011)] show considerably higher cholinergic than adrenergic tones; the relative importance of the respective system varies greatly among species, and, within the same specie, with developmental stage and sex. Acetylcholine (ACh) negatively modulates HR and cardiac contractility [for extensive review, see (Vornanen 2017)]. However, its effects are variable, being sometimes biphasic [*A. anguilla*: (Imbrogno et al. 2001)], or different between atrium and ventricle [*Godus morhua*: (Holmgren 1977)]. In the isolated and perfused goldfish heart, exogenous ACh decreases both HR and contractility (Cameron and Brown 1981; Cameron 1979). The response is abolished by atropine, suggesting the activation of muscarinic receptors. The ACh-dependent reduction of contractility is sensitive to temperature. It disappears in goldfish exposed to cold (5–8 °C) (Perrine and Georges 1978), showing similarities with the response reported in cold-acclimated eel *A. anguilla* (Seibert 1979).

The adrenergic tone is mediated by catecholamines (CAs), typically noradrenaline and adrenaline, released from adrenergic nerve terminals or reaching the heart via circulation. However, in several poikilotherms, including cyclostomes, elasmobranchs and some teleost, the peripheral nerves are replaced by aggregates of CAs-containing chromaffin cells, which represent components of the diffuse neuroendocrine tissue (Burnstock 1969). It is generally assumed that in teleosts, under resting conditions, plasma CAs levels are low and the nervous system plays a major tonic role on the heart (Axelsson 1988). However, in response to physical and environmental stressors (e.g., exhaustive exercise, hypoxia, acidosis), plasma CAs levels rapidly increase and, together with CAs released by nerve terminals and cardiac chromaffin cells, exert cardiac, vascular and respiratory responses that contribute to alleviate stress-induced detrimental effects (Farrell et al. 1986). Adrenergic stimulation, mediated by α- and β-adrenoreceptors (ARs) associated with both the pacemaker and the working myocardium, positively affects HR in different species [e.g., *A. anguilla* (Pennec and Le Bras 1984); *Oncorhynchus mykiss* (Graham and Farrell 1989)], and slightly improves the Frank–Starling response in the trout (Farrell et al. 1986). Information on the mechanisms underlying the humoral and neural CAs modulation of excitability in teleost cardiac pacemaker remains elusive. It has been proposed that, as in mammals, it could be related to changes in the density of sarcolemmal ion currents (I_f_ and I_CaL_), and/or to the cAMP-dependent modulation of molecular operators involved in the rate of Ca^2+^ cycling through the SR (e.g., L-type Ca2 + channels, RyRs, HCN channels) (Vornanen 2017). Notably, in the goldfish heart, HCN4 channels, which in mammals are responsible for the pacemaker current, are present in the pacemaker region, close to numerous axons and varicosities (Newton et al. 2014). This is similar to that described in zebrafish (Stoyek et al. 2015), and represents a local neural circuit for autonomic modulation of pacemaker discharge rate (Newton et al. 2014).

The stimulation of the isolated goldfish heart with sympathomimetic agonists (e.g. isoproterenol) elicits a positive chronotropism (Cameron and Brown 1981) which is abolished by the β-blocker, propranolol, but not by the α-adrenergic blocking agent phentolamine, thus suggesting a β-ARs activation (Cameron and Brown 1981). Data in several teleost species indicate that β-ARs subtypes are present in the heart [β1-AR: medaka (Kawasaki et al. 2008), zebrafish (Steele et al. 2011), fathead minnow (Giltrow et al. 2011); β2-AR: rainbow trout (Gamperl et al. 1994; Nickerson et al. 2001); fathead minnow (Giltrow et al. 2011), sockeye salmon (Goulding and Farrell 2016); β3-AR: rainbow trout (Petersen et al. 2013), fathead minnow (Giltrow et al. 2011), eel (Imbrogno et al. 2006), common carp and channel catfish (Petersen et al. 2015)]. In the goldfish, information on this issue is scarce. However, recent molecular and physio-pharmacological data show the expression of a functional β3-AR subtype (Leo et al. 2019). Under basal conditions, the stimulation of this receptor with BRL_37344_, whose action as β3-AR agonist is largely recognized, positively affects the goldfish heart performance via the adenylate cyclase/cAMP-dependent pathway (Fig. 2C). This effect is abolished by the specific β3-AR antagonist, SR_59230A_, but not by α/β1/β2-AR inhibitors. Very few studies documented in teleost the role of the β3-AR in the control of the cardiac function. Like in goldfish, in the channel catfish, the effects of BRL_37344_ stimulation are associated with changes in contractility and HR (Petersen et al. 2015). Conversely, in the rainbow trout (Petersen et al. 2013), common carp (Petersen et al. 2015), and freshwater European eel (Imbrogno et al. 2006), β3-AR stimulation is associated with a significant reduction of *V*s. These differences suggest a species-specific pattern of β3-ARs-dependent control of the teleost heart function which conceivably reflects the involvement of either Gi- or Gs-mediated pathways [reviewed in (Imbrogno et al. 2015)]. In fish, as in mammals, cardiac β3-ARs are generally coupled to Gi/o and to a transduction mechanism that, through the involvement of NO, modulates Ca^2+^ transients (Gauthier et al. 1998; Kitamura et al. 2000). However, several lines of evidence suggest a β3-ARs-dependent modulation of contractility associated with a Gs-dependent activation of adenylate cyclase (AC) and the consequent cAMP generation (Kohout et al. 2001; Skeberdis et al. 2008). Results obtained in goldfish support this last hypothesis. Notably, β3-ARs are cardioprotective in response to exercise, heart failure and ischaemia/reperfusion (I/R) injury (Balligand 2016; Niu et al. 2014; Salie et al. 2019). In agreement with their protective role, the β3-AR-dependent cardiostimulation observed in the isolated and perfused goldfish heart was proposed to contribute to preserve the performance under conditions of low O_2_ (Leo et al. 2019) (see below).

### Extrinsic regulation: the influence of cardioactive peptides

The long-established concept of a dual autonomic (sympathetic and parasympathetic) control of the fish heart has been recently enriched by the identification of nervous fibres releasing a variety of neurotransmitters, neuromodulators and peptides (Zaccone et al. 2010; Nilsson and Holmgren 1992; Stoyek et al. 2015). Together with both endocrine (circulating and intracardiac hormones) and autacoid (locally generated substances) signals, they regulate cardiac hemodynamics to meet animal physiological requirements [see for example (Gamperl and Farrell 2004; Amelio et al. 2013; Imbrogno and Cerra 2017)]. The coordinated/integrated expression and activity of these regulatory substances potentiates the ability of the heart of sensing, interpreting and responding to short-, medium- and long-term challenges deriving from internal and external environments (Tota et al. 2010). In the last decades, a proliferation of data from comparative research revealed that many well-recognized mammalian modulatory peptides have evolved in non-mammalian vertebrates as early regulators of cardiac molecular networks. In poikilotherms that routinely experience extreme ambient fluctuations, this neuro-endocrine control of the heart exerts a powerful protection of the organ in terms of function and survival, thus improving the success of the organism. This is the reason why the heart of vertebrates like fish and amphibians, also thanks to its relatively simple design and morpho-functional arrangement, can be easily used to investigate how cardiac neuro-humoral networks intertwine and interact in coordinating complex mechanisms and processes, whose importance is well recognized in mammals (Tota et al. 2010). In this field, recent advances place extraordinary value on the goldfish heart. Data from Newton et al. (2014) on the innervation of the goldfish heart, and the growing contribute of our research group on the humoral modulation of the goldfish cardiac performance (Mazza et al. 2015, 2019; Imbrogno et al. 2017; Leo et al. 2019), clearly revealed that in this teleost various extrinsic and intrinsic signalling pathways contribute to maintain cardiac steady-state under basal conditions and environmental challenges.

Here following, recent advances in the goldfish concerning the influence of cardiac modulators, their receptors, and the downstream signalling pathways will be illustrated (Table 1).

**Table 1:** Effects and mechanism of action of various cardio-active peptides on the goldfish (*C. auratus*) heart performance under basal and hypoxic conditions

	Basal performance under normoxia	Basal performance under hypoxia	Major signalling	References
p-GluSerp	Negative inotropism (33 nM)	ND	β3-AR-Gi/o-NO-cGMP cascade	Imbrogno et al. (2017)
SELENOT	Positive inotropism (10^–10^ M)	Positive inotropism (10^–8^ M)	cAMP/PKA cascade, L-type calcium channels and SERCA2a pumps	Mazza et al. (2019)
Nesfatin-1	Positive inotropism (10^–11^ M)	ND	cAMP/PKA cascade, L-type calcium channels and SERCA2a pumps	Mazza et al. (2015)

*ND* Not Detected

#### Chromogranin-derived peptides

Chromogranin A (CgA), a glycoprotein belonging to the chromogranin/secretogranin family, is ubiquitous throughout the animal world, from invertebrates to mammals, remarking a notable phylogenetic conservation (Helle et al. 2018; Bartolomucci et al. 2011). Originally identified in the secretory granules of the adrenal chromaffin tissue (Winkler and Fischer-Colbrie 1992), it was later found in secretory cells of the nervous, endocrine and immune system, co-stored and co-secreted with CAs, several hormones and neuropeptides (Helle et al. 2007). The vertebrate heart is a site for CgA production and, at the same time, a target for the protein. Immunoreactive CgA cells are present in the cardiac conduction system and in atrial myoendocrine granules of rodents (Steiner et al. 1990; Weiergraber et al. 2000; Tota et al. 2014), in the ventricular myocardium of humans (Pieroni et al. 2007), and in the secretory granules of frog atrial myocytes (Krylova 2007). As discussed by Mazza et al. (2010), the above evidence is fundamental for defining the cardiovascular properties of this protein in vertebrates. CgA is a pro-hormone for many regulatory peptides, generated by tissue-specific proteolytic cleavage operated by proteases and pro-hormone convertases. These peptides include: the dysglycemic hormone pancreastatin, the vasodilator vasostatins (VS-1 and VS-2), the catecholamine release inhibitory peptide catestatin (CST), and the C-terminal Serpinin peptides (Serp and the pyroglutaminated form, p-GluSerp), recognized for their protection against oxidative stress [for extensive review, see (Corti et al. 2018; Tota et al. 2014; Pasqua et al. 2015)].

The teleost heart is sensitive to CgA stimulation. This has been shown in the isolated and perfused eel *A. anguilla* heart, in which VSs and CST stimulation induces a cardio inhibitor effect (Imbrogno et al. 2004, 2010; Tota et al. 2004; Mazza et al. 2007). By using the same approach, Imbrogno et al. (2017) described in the goldfish a dose-dependent reduction of myocardial contractility by exogenous p-GluSerp. This negative inotropism is blocked by the pre-treatment with the β3-AR inhibitor, SR_59230_. It also involves G_i/0_ proteins and a NO/cGMP/protein kinase G (PKG)-mediated pathway. Information concerning the putative effects of other CgA-derived peptides on the goldfish heart function is not available. However, the negative effect elicited by p-GluSerp is in line with the cardio-inhibition mediated by others CgA-derived peptides in the teleost eel *A. anguilla*, in which VSs (Imbrogno et al. 2004) and CST (Imbrogno et al. 2010) counteract the positive inotropism induced by β-adrenergic (ISO) stimulation (Imbrogno et al. 2004, 2010), indicative of a cardioprotection in response to excitatory stimuli (Tota et al. 2004).

#### Selenoprotein T

Selenoproteins are a family of proteins incorporating selenium in the form of selenocysteine (Sec), whose presence in the catalytic site confers oxido-reductase properties. 45 selenoproteins have been detected in mammalian and non-mammalian vertebrates, including bony fishes, which show the largest selenoproteome (Mariotti et al. 2012). Among selenoproteins, selenoprotein T (SelT or SELENOT), a thioredoxin-like enzyme of the endoplasmic reticulum, is proposed as a cardiac modulator in mammals, able to elicit cardioprotection against ischemia–reperfusion (IR) injury (Rocca et al. 2019a). In the goldfish, three SELENOT paralogs (gfSelT1a, gfSelT1b and gfSelT2) are present in different tissues, including the heart (Chen et al. 2017). Contrarily to mammals, in which SELENOT expression is high during ontogenesis and progressively disappears in adult tissues (Tanguy et al. 2011), the goldfish heart expresses SELENOT also in the adulthood, although at lower levels then in the juvenile (Mazza et al. 2019). This is common in teleost (Mazza et al. 2019), and is probably related to the high capacity of the adult fish heart to grow throughout life (Cerra et al. 2004), or in response to stress stimuli (Wills et al. 2008). Of note, in the goldfish, exposure to low O_2_ significantly increased cardiac SELENOT expression (Mazza et al. 2019). Moreover, in the isolated and perfused goldfish heart, PSELT, a 43–52 SELENOT-derived peptide, reduces hypoxia-dependent nitrosative stress (Mazza et al. 2019). This confirms the oxido-reductase properties attributed to selenoproteins in mammalian and non-mammalian vertebrates, and is in line with the cardioprotection against ischemic injury reported in the rat (Rocca et al. 2018a). In the isolated and perfused goldfish heart, exogenous PSELT significantly affects myocardial performance (i.e., the ability of the heart to perform its functions) by increasing contractility (i.e., the force generated by cardiac contraction at a given preload, afterload and heart rate) in a dose-dependent manner (Mazza et al. 2019). This is related to the activation of a cAMP/protein kinase A (PKA) pathway and involves L-type calcium channels and SERCA2a pumps. Interestingly, in the goldfish, an enhanced mechanical performance is crucial for maintaining functional and metabolic interactions between organs and tissues under hypoxia (Imbrogno et al. 2014). The positive inotropic effect induced by SELENOT, together with its ability to reduce hypoxia-dependent nitrosative stress, suggests a role in the mechanisms involved in the cardiac response of the goldfish to hypoxia.

#### Cardiac action of feeding-regulating factors: Nesfatin-1

Several peptides, first identified for functions other than cardiovascular control, are now recognized as important modulators of cardiac function. This is the case of feeding-regulating factors such as ghrelin, cholecystokinin, Neuropeptide Y, vasoactive intestinal peptide, Nesfatin-1, that, by acting centrally, via the autonomic nervous system, or directly, on cardiac and endothelial vascular cells, affect cardiovascular homeostasis.

In the last 2 decades, several feeding-regulating factors characterized by aminoacid sequence, structures and modes of action similar to their mammalian counterparts, have been isolated in the goldfish. Although several evidence indicates the expression of appetite regulatory peptides in different tissues of *C. auratus* [see for ref. (Blanco et al. 2018)], only VIP and Nesfatin-1 have been reported either to be present or to affect the cardiac function. VIP positive staining is present in the goldfish in the terminal axonal varicosities of the sinoatrial plexus (SAP) around intracardiac neurons (ICN) somata (Newton et al. 2014); however, no information is available on the possibility that VIP affects the goldfish cardiac function.

Nesfatin-1 is a hypothalamic 82-aa peptide, proteolytically derived from the larger precursor Nucleobindin 2 (NUCB2) (Oh et al. 2006). In the goldfish, nesfatin-1-like-immunoreactive cells are present in the hypothalamus, within the nucleus lateralis tuberis (NLT), and in the anterior intestine, J-loop (Gonzalez et al. 2010). Nesfatin-1 expression has been also found in gills and skeletal muscle (Mazza et al. 2015). Different from mammals (Angelone et al. 2013), the goldfish heart does not express Nesfatin-1 (Mazza et al. 2015), although low levels of NUCB2 mRNA expression are present in cardiac extracts (Gonzalez et al. 2010). This suggests that NUCB2 is not processed into Nesfatin-1, or alternatively that the amount of Nesfatin-1 generated by NUCB2 cleavage in the goldfish heart is below the detectable range. Of note, physiological studies performed using an ex vivo working goldfish heart preparation revealed a remarkable sensitivity to the peptide. Mazza et al. (2015) showed that the exogenous Nesfatin-1 induces a dose-dependent increase of myocardial contractility via a mechanism that involves a cAMP/PKA pathway, L-type calcium channels, and SERCA2a pumps. Previous evidence in mammals proposed a central cardiovascular action for Nesfatin-1, since hypertension and tachycardia has been observed in response to intracerebroventricular administration of the peptide (Mimee et al. 2012). The goldfish heart preparation used by Mazza et al. to analyse the cardiac activity of Nesfatin-1 is free of innervation. Accordingly, it can be assumed that the peptide may act directly on the heart. However, it is possible that, as in mammals, in in vivo animals, the direct cardiac effects of Nesfatin-1 may be additive and/or alternative to a central action on brain regions responsible for the cardiovascular control. In the goldfish, Nesfatin-1-induced cardiostimulation correlates to an increased phosphorylation of extracellular signal-regulated kinases1/2 (ERK1/2), a feature that in mammals has been associated to a cardioprotection against stress challenges (e.g., ischemia) (Rocca et al. 2018b; Darling et al. 2005). This allows to hypothesize that, in an organism characterized by a striking capacity to face environmental challenges, such as the goldfish, the cardiostimulation induced by Nesfatin-1, together with the activation of the protective ERK1/2 cascade, may contribute to the endogenous mechanisms of stress resistance (Mazza et al. 2015).

### The paracrine/autocrine modulation of the goldfish heart performance: a role for NO and its metabolites

**Fig. 4:**
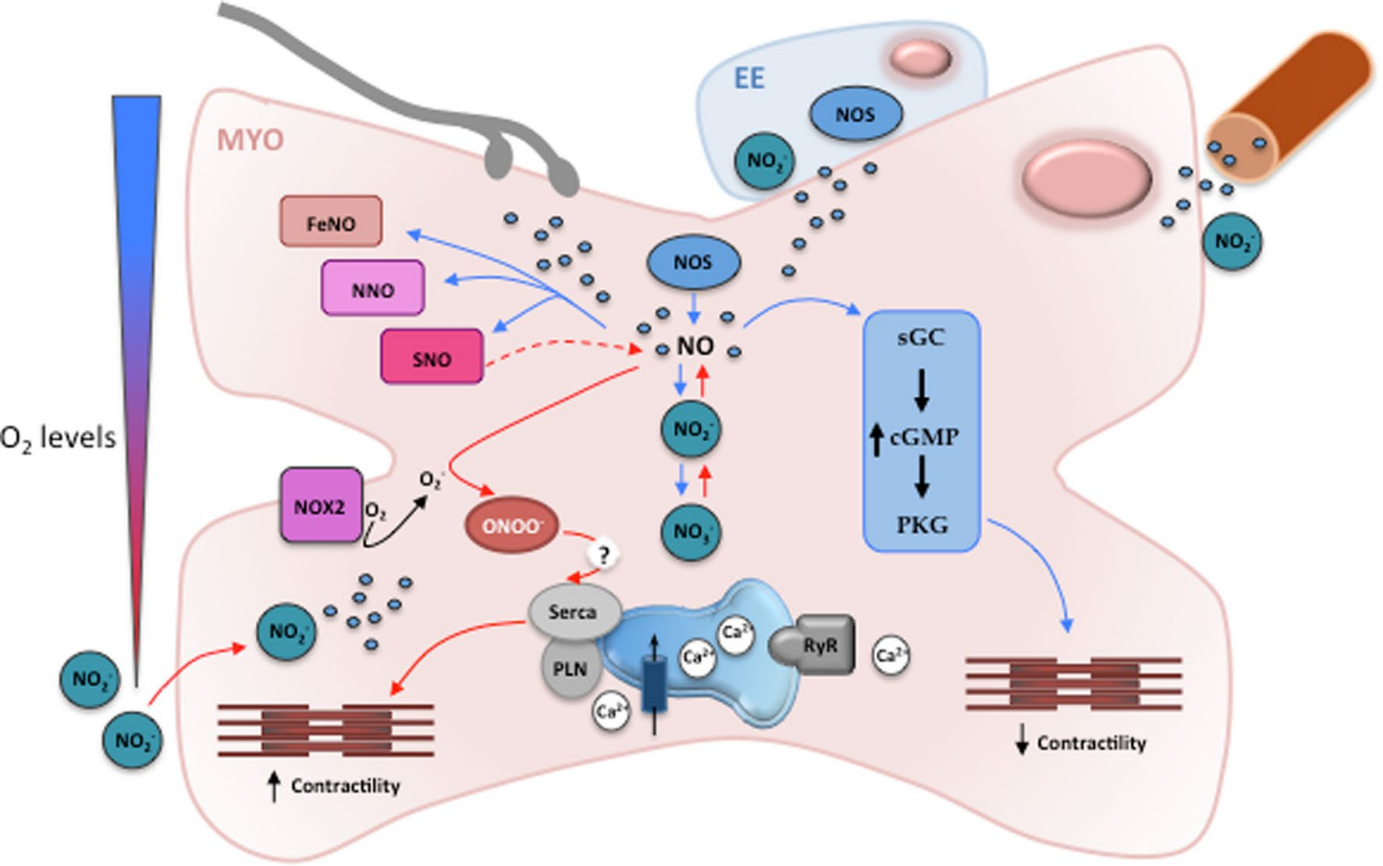
Schematic representation of the role of NO and its metabolites in the paracrine/autocrine modulation of the goldfish heart performance under normoxic (blue arrows) and hypoxic (red arrows) conditions. In the goldfish heart positive staining for NOS isoenzymes have been detected at the level of the vascular endothelium, in the endocardium endothelium (EE), and in cardiomyocytes (MYO), as well as in intracardiac neurons and axons in the sinoatrial plexus. Under normoxia NOS-produced NO may affect myocardial function via the activation of classical (cGMP dependent pathways) and non-classical mechanisms (protein nitrosylation). Under acute hypoxia, an increased NOS expression together with an augmented nitrite (NO_2_^−^) conversion to NO, contributes in increasing intracellular NO levels and this is associated with the hypoxia-dependent increase of myocardial contractility. Protein denitrosylation and protein nitration have been also proposed as regulatory mechanisms in the response of the goldfish heart to hypoxia. *NOS* nitric oxide synthase, *Nox2* NADPH oxidase-2, *ONOO*^*−*^ peroxynitrite, *sGC* soluble guanylyl-cyclase, *PKG* protein kinase G, *Serca* sarcoplasmic reticulum Ca^2+^ ATPase, *PLN* phospholamban, *RyR* ryanodine receptor. Modified from (Imbrogno et al. 2018)

NO, the first gas identified as a biologically relevant molecule (Ignarro et al. 1987), is a major autocrine–paracrine organizer of the basal cardiac physiology and a key coordinator/integrator of physically and chemically activated intracellular pathways [for extensive review, see (Imbrogno and Cerra 2017; Imbrogno et al. 2018)]. In almost all animal tissue, NO is produced from L-arginine by Nitric Oxide Synthase (NOS) enzymes [i.e., constitutive endothelial (e)NOS and neuronal (n)NOS, and inducible (i)NOS] in a process which requires the presence of molecular O_2_ and NADPH as cofactors. NO has a short half-life (from 2 ms to 2 s) (Thomas et al. 2001); it is rapidly metabolized to more stable metabolites, such as nitrite, nitrate, iron-nitrosyl, S-nitroso and N-nitroso compounds (Hansen and Jensen 2010) (Fig. 4). NO metabolites, particularly nitrite, are widely used as markers of NOS activity (Kleinbongard et al. 2003). The heart represents a major site of NO production, as well as a target of its actions. NO is generated by myocardial and non-myocardial tissues (i.e., vascular and endocardial endothelial cells, interstitial cells, coronary vessels and myocardial neurons), and elicits a paracrine-autocrine control of beat-to-beat, short-term and long-term cardiac responses [for extensive review see (Imbrogno et al. 2018)].

The goldfish heart shows positive staining for NOS isoenzymes at the level of the vascular endothelium, in the EE and in cardiomyocytes (Garofalo et al. 2012). nNOS signals are also present in intracardiac neurons and axons in the sinoatrial plexus of the goldfish heart (Newton et al. 2014) (Fig. 4). In the isolated and perfused goldfish heart, the use of either NOS enzyme inhibitors (e.g., L-NMMA) or NO donors (e.g., GSNO) showed that NO tonically depresses the basal mechanical performance, while it enhances the Frank–Starling response (Imbrogno et al. 2014). Both regulations involve cGMP-mediated pathways which, possibly by modulating different Ca^2+^-dependent and Ca^2+^-independent mechanisms, can either depress contractility or accelerate relaxation to enhance the sensitivity to the Frank–Starling response (Imbrogno et al. 2014).

A significant cardiac reservoir of NO is represented by nitrite. Under particular conditions, such as hypoxia and acidosis, nitrite generate NO through non-enzymatic and enzymatic pathways, including acidic disproportionation, and reduction via endogenous metal-containing enzymes, such as deoxygenated Hb, myoglobin, neuroglobin, xanthine oxidoreductase and carbonic anhydrase [references in (Jensen 2009)]. Circulating NO_2_^−^ can act as a signalling molecule by itself (Gladwin et al. 2006), or as a vascular endocrine NO reservoir (Kim-Shapiro et al. 2006). As in mammals (Cosby et al. 2003; Duranski et al. 2005; Webb et al. 2004), in fish, it represents a key modulator of many biological processes [e.g. cardiovascular function, cytoprotection (Pedersen et al. 2010)]. In the goldfish exposed to normal O_2_ levels, as in other hypoxia-tolerant species, plasma levels of nitrite are slightly higher than those measured in hypoxia-sensitive animals [ref. in (Fago and Jensen 2015)]. This may reflect a higher NOS activity under normoxia, as well as an additional water nitrite up-take through the gills (Jensen and Hansen 2011) due to an active chloride uptake mechanism (Williams and Eddy 1986). Of note, the goldfish, as the crucian carp, maintains tissue nitrite concentration during hypoxia and increases it in the heart during deep hypoxia and anoxia (Hansen et al. 2016a; Hansen and Jensen 2010; Sandvik et al. 2012). This occurs at the expenses of extracellular nitrite concentration. It has been proposed that the intracellular binding of nitrite to proteins keeps low the cytosolic concentration of free nitrite thus favouring inward diffusion (Hansen and Jensen 2010). In addition, *Carassius* members, as many other vertebrates, possess a tissue nitrate reductase activity (usually mediated by xanthine oxidoreductase) that, by reducing nitrate to nitrite, enriches internal nitrite pool, thus securing NO availability during deep hypoxia (Hansen et al. 2016b). This is important to maintain internal nitrite levels for securing a NO source when the NOS activity is compromised by limited O_2_.

## Cardiac adaptation to environmental challenges

### Hypoxia

Cyprinids fish, as the goldfish and the crucian carp, together with some species of turtles, are the most hypoxia-tolerant vertebrates, surviving complete O_2_ deprivation for long periods (Bickler and Buck 2007). Since the end of the 1970s, the scientific interest in understanding the physiological mechanisms underlying the ability of these anaerobic champions to survive without O_2_ significantly increased. In a landmark paper, Shoubridge and Hochachka (1980) reported the exceptional discovery that the goldfish is able to tolerate prolonged O_2_ absence by using large glycogen stores to generate ethanol as a by-product of energy metabolism, thus avoiding acidosis (Shoubridge and Hochachka 1980). Only recently, Fagernes et al. (2017) demonstrated that this is due to the presence in *Carassius* (*C. carassius* and *C. auratus*) of an alternative E1 pyruvate dehydrogenase enzyme, one of the catalytic component of the pyruvate dehydrogenase complex (PDHc). As suggested by the authors, following whole-genome duplication events, additional gene copies of E1 subunits have evolved into a pyruvate decarboxylase which, under anoxia, catalyses the conversion of pyruvate to acetaldehyde. Then, a muscle-specific alcohol dehydrogenase converts acetaldehyde into ethanol (Fagernes et al. 2017), which is rapidly eliminated through the gills.

A preserved heart performance is essential for improving metabolic and functional cooperation among single organs, thus providing anoxia resistance to the whole fish organism (Gattuso et al. 2018). Notably, under hypoxia, the goldfish heart improves its cardiac performance (Imbrogno et al. 2014). When perfused with a hypoxic medium (2.5 ± 0.3 mg O_2_ L^−1^; concentration of O_2_ reaching the isolated heart ~ 1.5 mg O_2_ L^−1^), the ex vivo isolated working heart of *C. auratus* exhibits an enhanced basal mechanical performance, with *V*s increasing in a time-dependent manner (Imbrogno et al. 2014). This is particularly evident in the response to preload increases in which the maximum *V*s is reached at input pressures lower than the normoxic heart (Fig. 3) (Imbrogno et al. 2014). This feature, which at the moment seems to be a prerogative of the goldfish, is proposed as a mechanism to properly support organ perfusion, thus preventing tissue intoxication (Imbrogno et al. 2014). The molecular bases underlying the hypoxia-dependent increase of the goldfish myocardial contractility are unclear. Recently, it was observed that β3-ARs expression increases in isolated and perfused goldfish hearts exposed to acute hypoxia (Leo et al. 2019), and that the presence of SR_59230A_, a selective inhibitor of β3-ARs, abolishes the hypoxia-dependent increase of myocardial contractility (Leo et al. 2019). In mammals, β3-ARs activation is related to cardioprotection (Balligand 2016). In addition, as recently reported by Dal Monte and collaborators, β3-AR expression markedly increases under hypoxia (1% O_2_) in ex vivo retinal explants (Dal Monte et al. 2013). The above evidence suggests that in the goldfish, as in mammals, cardiac β3-ARs participate in the protective mechanism activated under low O_2_. In contrast, in the hypoxia-sensitive trout *O. mykiss*, acclimation to moderate chronic hypoxia (≈40% air saturation) correlates with a loss of cardiac β3-ARs associated to a reduced heart pumping capacity (Motyka et al. 2017). This suggests that the role of cardiac β3-ARs under hypoxia correlates with the species-specific ability to face low oxygen (see hypoxia-sensitive trout vs. hypoxia-tolerant goldfish).

As discussed above, the role of NO and its metabolites in the control of the cardiac function under O_2_ deprivation is well established [for extensive review see (Imbrogno et al. 2018; Schulz et al. 2004)]. In the hypoxic goldfish heart, NO inhibits mitochondrial respiration with no effect on contractility (Pedersen et al. 2010). This increases myocardial efficiency (i.e., the force generated per O_2_ consumed), thus contributing to maintain myocardial function (Stecyk et al. 2004). In line with these observations, studies from our lab revealed in the isolated goldfish heart that acute hypoxia (~ 1.5 mg O_2_ L^−1^) is accompanied by an increased myocardial NOS expression (Imbrogno et al. 2014), and that the NOS-derived NO is crucial for the hypoxia-dependent increase of myocardial contractility, typical of this teleost (Filice et al. 2020b). This is supported by the hypoxia-induced activation of the phosphatidylinositol-3 kinase (PI3-K)/protein kinase B (Akt) pathway, a well-known player in the NOS-dependent NO production (Carrillo-Sepulveda et al. 2010), and SERCA2a pumps modulation, whose inhibition under hypoxia significantly reduced the time-course increase of the goldfish heart performance (Filice et al. 2020b). A modulation of SERCA2a pumps by nitration events has been suggested contributing to the high resistance of the goldfish heart to conditions of reduced oxygen (Filice et al. 2020b). Of note, in the goldfish, the hypoxia-induced increase in NO production also activates cardiac sarcolemmal KATP channels via a cGMP-dependent pathway (Chen et al. 2005), a response that, similarly to the KATP-dependent cardioprotection activated in the ischemic mammalian myocardium (Noma 1983), may enhance the hypoxia tolerance of this species (Cameron et al. 2003).

The goldfish can maintain routine metabolic rates at severely hypoxic PwO_2_ values (from 20 to 1.3 kPa), depressing it only in response to anoxia (between 0.67 and 0 kPa) (Regan et al. 2017). Moreover, in this species, hypoxia exposure results in a temporary increase of lactate concentration, which goes back, within a few hours, to values similar to normoxia (Regan et al. 2017). Accordingly, a reduced LDH activity was recently observed in homogenates of goldfish heart exposed to acute hypoxia (~ 1.5 mg O_2_ L^−1^) (Imbrogno et al. 2019a). This suggests that the enhancement of myocardial contractility observed in the goldfish in response to low O_2_ is associated with a low accumulation of cardiac lactate. In addition, authors reported a parallel slight reduction in pyruvate levels (Imbrogno et al. 2019a): this implies the activation of alternative metabolic pathways (e.g., gluconeogenesis) which, by allowing a reutilization of the first product of the anaerobic glycolysis, enhance anaerobic ATP yield and minimize metabolic acidosis. Mass spectrometry-based proteomic analysis led to the identification of two isoforms of fructose-bisphosphate aldolase differently expressed in homogenates of goldfish heart exposed to normoxic or hypoxic medium (Imbrogno et al. 2019a). Particularly, Aldolase C, mainly effective in glycolysis (Penhoet et al. 1969; Penhoet and Rutter 1971), has been found in the normoxic heart, while aldolase B, which has evolved to have a role in gluconeogenesis (Penhoet and Rutter 1971; Penhoet et al. 1969), has been mainly revealed in the hypoxic counterpart (Imbrogno et al. 2019a). This supports the possibility that in the goldfish exposed to reduced O_2_, a tightly modulation of the aldolase enzyme isoforms may finely regulate glycolytic *vs.* gluconeogenic flux, thus minimizing deleterious waste accumulation also mitigating the negative consequences of a low hypoxia-dependent ATP production.

**Fig. 5:**
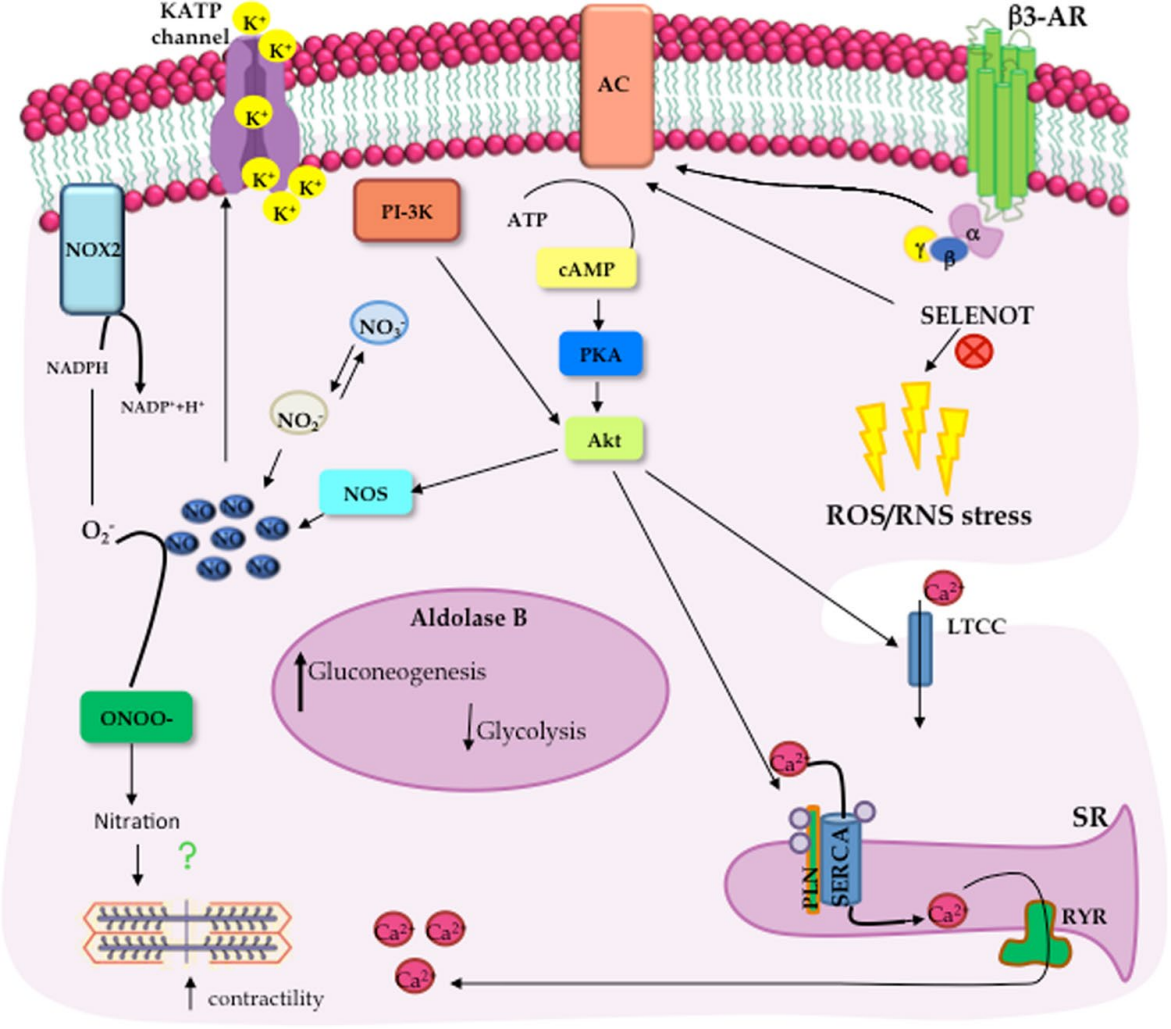
Simplified overview of intracellular mechanisms activated in the goldfish heart under hypoxia. Hypoxia exposure increases β3-ARs cardiac expression which activates a cAMP-dependent pathway. Under low oxygen, intracellular NO levels increase as a consequence of both augmented NOS expression and nitrite (NO_2_^−^) conversion. In parallel, the activation of the PI3-K/Akt pathway and the Nox2 enzyme cause the simultaneous generation of NO and O_2_^−^, respectively, thus contributing to protein nitration. The role of SELENOT to lessening the hypoxia-dependent nitrosative stress is showed. The hypothesis of a tight modulation of the aldolase enzyme isoforms that, by finely regulating glycolytic vs. gluconeogenic flux minimizes deleterious waste accumulation, is also reported. *NOS* nitric oxide synthase, *Nox2* NADPH oxidase-2, *ONOO*^*−*^ peroxynitrite, *AC* Adenylate cyclase, *PI-3K* Phosphatidylinositol-3 kinase, *PKA* protein kinase A, *Akt* protein kinase B, *LTCC* L-type Ca^2+^ channel, *Serca* sarcoplasmic reticulum Ca^2+^ ATPase, *PLN* phospholamban, *RyR* ryanodine receptor, *ROS/RNS* reactive oxygen species/reactive nitrogen species

An overview of the intracellular mechanisms activated in the goldfish heart under hypoxia is depicted in Fig. 5.

The information above summarized reveals novel aspects of the still-unresolved mechanisms that sustain the elevated tolerance to hypoxia typical of several vertebrates. It also proposes the goldfish heart as an experimental tool not only to enhance basic knowledge, but also to complement results deriving from more traditional models to explore the fundamental molecular mechanisms of cardiac plasticity in vertebrates, as well as their evolutionary history.

### Temperature

Ectothermic fishes living in temperate environments experience both acute and chronic fluctuations in ambient temperature that, by affecting body homeostasis, may elicit adverse effects on the heart function. Goldfish exhibits striking capacity of coping with a wide range of ambient temperatures and this contributed to the wide zoogeographic distribution and success of this species (Ford and Beitinger 2005). The pivotal studies of Fry et al. (1942), which first proposed the now well established thermal polygon for the goldfish, and the following studies of Ford and Beitinger (2005) concerning the tolerance of this teleost to dynamic temperature changes demonstrated the remarkable ability to survive to temperatures between 0° and 41 °C, as well as to short-term exposures at values close to 44 °C (Ford and Beitinger 2005). In addition, a study performed by Ferreira et al. (2014) showed that, in the goldfish, warm acclimation increases the upper thermal tolerance and can reset maximum HR to a lower rate for a common test temperature. Nevertheless, evidence of goldfish cardiac adaptation to thermal changes is scarce and mainly concerns acclimation to low temperatures. In non-polar fish, acute or chronic decreases in environmental temperatures normally result in a decrease in the resting HR (Keen et al. 2017). Two potential extreme strategies are proposed to face cold-induced bradycardia: an increased heart size, which, in turn, increasing resting *V*s*,* could maintain CO, and an increased HR with concomitant decrease in time to relaxation (Driedzic et al. 1996; Keen et al. 2017). Both responses have been reported in the goldfish. Tsukuda et al. (1985) demonstrated that the heart of goldfish acclimated to low temperature (10 °C) has a higher weight than its warm-acclimated counterpart (25 °C). Moreover, spontaneously contracting isolated heart preparations from cold-acclimated fish had higher frequencies and amplitudes of contraction with respect to warm-acclimated animals (Tsukuda 1990). In teleosts, changes in HR and cardiac force in part correlate to the direct effects of temperature on ion channels and pumps which trigger excitation–contraction coupling (Shiels et al. 2003; Vornanen et al. 2014). Ganim et al. (1998) proposed a role for ATP-sensitive potassium channel (KATP) in the mechanisms promoting cardiac tolerance to low temperature in the goldfish. In fish acclimated to 7 °C, they observed an augmented activity of cardiac KATP channels, expressed as an increased channel mean open-time and overall open-state probability (Ganim et al. 1998). Sustained KATP activation also shortened action potential duration, and this is proposed to limit Ca^2+^ influx (Sauviat et al. 1991). Notably, a decreased Ca^2+^ ion permeability is suggested to confer an energetic advantage, promoting survival in cold environments and helping to match cell metabolic requirements with ATP availability (Ganim et al. 1998). In addition, KATP channels in cardiac cells from goldfish acclimated at 7 °C are less sensitive to ATP-inhibition, thus reflecting a functional adaptation to promote tolerance of low temperatures (Ganim et al. 1998). These data support the possibility that in the goldfish, like in mammals, KATP channels are protective in the heart during metabolic stress. They also propose KATP chronic activation as a mechanism that, in cold-tolerant animals, promotes survival at low body temperatures.

### Exposure to pollutants

Rapid growth of human populations, technological advancement, and widespread use of chemicals in industry and consumer products, result in a rapid diffusion of innumerable pollutants and environmental toxins (Franzellitti et al. 2019). The occurrence of such contamination in water environments profoundly damages wildlife and fish health by affecting reproduction, lifespan, and embryonic and larval development (Bambino and Chu 2017). They may induce neurotoxicity, oxidative damages, and genotoxicity and disturb the immune system and organ integrity (Wang et al. 2020). At the same time, some aquatic organisms thrive and reproduce also in the presence of multiple toxic compounds since they possess endogenous defence mechanisms, showing a multi-drug-resistance like behaviour (Bard 2000). The analysis of the large variety of responses allows to describe the species-specific biological events induced by water pollutants. At the same time, it opens the possibility to test the range of tolerability not only of single animal species, but also of the whole environment. In this context, the amazing capability of the goldfish to physiologically adapt to different challenges makes it a good bioassay for studies aimed to provide information of environmental and conservation concerns. For this reason, a growing number of researches use the goldfish to assess the effects of water contaminants. Available data reveal that exposure to water born toxicants induce alterations in thermal tolerance (Gandar et al. 2016, 2017; Jacquin et al. 2019), reproductive behaviour (Golshan et al. 2014, 2015; Wang et al. 2019; Zhang et al. 2018), oxidative balance (Xu et al. 2015; Zheng et al. 2014; Gandar et al. 2017), tissue morphology (Husak et al. 2014; Velma and Tchounwou 2010; Zhang et al. 2005), and organ function (Filice et al. 2020a; Kubrak et al. 2012). Also, the cardiac function of the goldfish is affected by pollutants. For example, high concentrations of microplastics increase HR in goldfish larvae (Yang et al. 2020); exposure to the lambda-cyhalothrin (LCT) insecticide influences cardiac levels of metabolites such as lactate, choline, taurine, phosphocreatine, with alteration of oxidative status and disturbed energy metabolisms (Li et al. 2014). Recently, also Bisphenol A (BPA), a common environmental pollutant used in polycarbonate plastics and epoxy resins, was found to severely affect the cardiac function of adult goldfish (Filice et al. 2020a). High BPA concentrations (25 μM) decrease HR, alter the basal performance (i.e. a higher preload pressure to reach the physiological baseline cardiac output) and impair the Frank–Starling response. They also induce an enhancement of cardiosomatic indices and ventricular muscularity, together with an increase of focal areas of collagen deposition. BPA also alters the oxidative status and, at high doses and long times of exposure, strongly compromises the expression of stress (HSPs) and proapoptotic (Bax and Cytochrome C) markers (Filice et al. 2020a).

## Cardiac remodelling and regenerative potential

In fish, the adult heart retains an extraordinary ability to grow in response to several physiological stimuli, including ontogenetic growth (Cerra et al. 2004), environmental challenges, increased demand associated with training/exercise activity and sexual maturation (Gamperl and Farrell 2004). In teleost, cardiac morpho-functional remodelling has also been reported in the adult as a response to humoral stimulation [AngII: (Filice et al. 2017, 2021; Imbrogno et al. 2013); cortisol: (Johansen et al. 2017)] and after exposure to environmental pollutants (Filice et al. 2020a). Remodelling reaches its maximal expression in the astonishing capacity shown by several species to fully regenerate the injured heart. Examples are the danionins zebrafish and giant danio (Lafontant et al. 2012; Poss et al. 2002). In zebrafish, following resection of the ventricular apex, the injured area is completely replaced by proliferating myocardiocytes with a reduced development of fibrosis, and a robust ventricular regeneration (Poss et al. 2002). This ability is a trait also common to the adult goldfish (Grivas et al. 2014). As in zebrafish and the giant danio, in the goldfish ventricle, a triad of temporally overlapping processes, i.e., inflammation, collagen accumulation and angiogenesis, characterizes the repair and regeneration of small cauterized areas (Grivas et al. 2014). In fish, as in mammals, the early inflammatory response is crucial for promoting correct tissue repair (Huang et al. 2013; Sun et al. 2009). Of relevance, in the goldfish, the inflammatory cell population includes melanomacrophages, whose presence in the fish heart had never been shown before (Grivas et al. 2014). Melanomacrophages are found in a number of cold-blooded species. In Osteichthyes, these cells form a cluster, i.e., the melanomacrophage centres (MMCs), predominant in the stroma of hematopoietic and lymphoid tissues (Stosik et al. 2019). Histological analyses suggested that MMCs are structurally similar to the mammalian germinal centres (GC), leading to the hypothesis that, as in mammals, also in poikilotherms, MMCs play a role in the humoral adaptive immune response (Steinel and Bolnick 2017). Melanomacrophages have been previously detected in the kidney and the spleen of *C. auratus*, where they act as phagocytes (Herraez and Zapata 1986, 1991). The presence of these cells in the heart during repair and regeneration suggest that, in this teleost, they play a role in injury response and cardiac remodelling (Grivas et al. 2014). Together with the inflammatory response, a marked accumulation of collagen fibres, an enhancement of connective tissue and neovascularization occurs in the transition zone between healthy and injured myocardium and in adjacent sub-epicardial regions (Grivas et al. 2014). Of note, a similar pattern characterizes the structural rearrangement observed in the goldfish heart in response to BPA-induced stress (Filice et al. 2020a). This suggests that, regardless of the stimulus activating the event, these processes are common mechanisms which in this teleost sustain cardiac remodelling. In the goldfish ventricle, the injured area is populated by partially differentiated or dedifferentiated cardiomyocytes and this indicates that cell proliferation contributes to reconstitute the ablated myocardial tissue (Grivas et al. 2014).

The above results allow to add the goldfish to the list of teleost models for studying the mechanisms of cardiac injury, repair, and regeneration. In a translational perspective, keeping in mind the still urgent problem of repairing the damaged mammalian heart, this may represent a unique opportunity.

## Conclusions

As illustrated in this review, the growing literature on the goldfish is progressively revealing the potential of this teleost in many fields of cardiac research. The ability to endure environmental extremes, associated with an extremely easy handling, the large commercial availability and low cost, the relatively short life, the possibility of genetic manipulation, and the relatively simple cardiac design, make the goldfish a valuable model for laboratories interested in exploring cardiac function, mechanisms of adaptation, and possibilities for repair after damages. The above advantages are accompanied by the recent sequencing of the goldfish whole genome (Chen et al. 2019). This represents an important resource also for analyses aimed at understanding the genetic basis of vertebrate cardiac development and evolution (Omori and Kon 2019). We hope that the advantages offered by the extremely flexible heart of this amazing vertebrate will cross the boundaries of animal physiology laboratories and will reach a larger research audience, with potential benefit also for mammalian-oriented studies.

## Data Availability

All data used in this study have been previously published.
